# Clinical trial data visualisation

**DOI:** 10.1186/1745-6215-16-S2-P187

**Published:** 2015-11-16

**Authors:** Cassie Gregson

**Affiliations:** 1 AstraZeneca, Alderley Park, UK

## 

Data collected throughout the duration of a clinical trial can amount to tens of thousands, or even hundreds of thousands of data points; which require expert interpretation and analysis to determine the efficacy, tolerability and safety profile of an investigational drug. Continuous monitoring and interpretation of these raw data are critical in maintaining patients’ safety. Realising this, however, has proved a significant challenge, due to the requirement to manually aggregate patient and population data to compile differing clinical data types (e.g. Adverse Events (AEs) and laboratory measurements) over multiple time-points. Additional data challenges are identified in the data formatting and presentation, which is required for successful and accurate interpretation. Furthermore, once a clinical trial has finished, analysis and interpretation of the validated data is mandatory.

In order to address the key data challenges, we have developed automated data integration and visualisation tools; REACT (REal-time Analytics for Clinical Trials) for on-going trials, and DETECT (Data Evaluation Tool for the End of Clinical Trial data) for finished trials. In this talk, REACT and DETECT will be presented to show how they provide an intuitive visual platform for data interpretation; enabling physicians to interact with the data, quickly view relationships between different clinical data and assess these data over time; both at a patient and population level. The result of which help us to keep our trial participants safe, improve our ability to efficiently make data-driven, scientific decisions and ultimately contribute to the development of medical treatments to improve the lives of patients.

